# J-Matrix time propagation of atomic hydrogen in attosecond fields

**DOI:** 10.1038/s41598-022-14706-9

**Published:** 2022-07-01

**Authors:** Rolf Gersbacher, John T. Broad

**Affiliations:** University of Applied Sciences Esslingen, Robert Bosch Str. 1, 73037 Göppingen, Germany

**Keywords:** Atomic and molecular physics, Atomic and molecular interactions with photons, Attosecond science

## Abstract

The J-Matrix approach for scattering is extended to the time-dependent Schrödinger equation (TDSE) for one electron atoms in external few cycle attosecond fields. To this purpose, the wave function is expanded in square integrable ($L^2$) Sturmian functions and an equation system for the transition amplitudes is established. Outside the interaction zone, boundary conditions are imposed at the border in the $L^2$ function space. These boundary conditions correspond to outgoing waves (Siegert states) and minimize reflections at the $L^2$ boundary grid. Outgoing wave behaviour in the asymptotic region is achieved by employing Pollaczek functions. The method enables the treatment of light - atom interactions within arbitrary external fields. Using a partial wave decomposition, the coupled differential equation system is solved by a Runge-Kutta method. As a proof of the method ionization processes of atomic hydrogen in half and few cycle attosecond fields are examined. The electron energy spectrum is calculated and the numerical implementation will be presented. Different forms of the interaction operator are considered and the convergence behaviour is discussed. Results are compared to other studies which use independent approaches like finite difference methods. Remarkable agreement is achieved even with strong field strengths of the electromagnetic field. It is demonstrated that expanding in $L^2$ functions and imposing boundary conditions at the limit in the $L^2$ function space can be an advantageous alternative to conventional propagation methods using complex absorbing potentials or complex scaling.

## Introduction

The availability of tailored electromagnetic fields in the attosecond range^1–3^ allows examining time-dependent phenomena that don’t appear in the long time limit. Carrier-envelope phase effects in ionization^4–6^ and recombination^7,8^ processes and the control of wave packets^9,10^ with combined UV and IR pulses^11,12^ can both be named as examples and even half cycle pulses in the attosecond range seem to be realizable^13^. On the theoretical side, describing ionization processes is a challanging task, because the wave function propagates without limit in space. The situation worsens with longer interaction times where the ejected particle can escape to infinity. In almost all cases, a partial wave expansion of the Schrödinger equation is used. The angular part is represented by spherical harmonics and the radial part is discretised; techniques like B-Splines^14^, finite difference methods^6,15^ or the discrete variable representation^16,17^ are employed. Strong fields and long interaction times lead to occupation of high partial waves. Hence, the radial position space above a given limit is disregarded or, alternatively, a limit is chosen which is so big that, throughout the whole time of propagation, it is not exceeded. Other approaches use Dirichlet to Neumann or Neumann to Dirichlet boundary conditions at some specified radial distance $r=R$ in the spatial domain to solve the TDSE or formulate boundary conditions via a convolution integral with the Feynman Propagator of the interaction free Schrödinger equation^18–21^. A further method to deal with limited radial distances is complex scaling. There, the wave function is rotated into the complex plane, which dampens the radial part and thereby reduces the radial extension^22^. Another possibility, which can find application with finite propagation radii or with a finite dimensional function space, is the introduction of a complex potential term localized at the boundary area of the position space or the function domain. This absorbs impinging waves to prevent interfering unphysical reflections that return into the interior zone^23^. For both methods, complex scaling as well as complex absorbing potential, no knowledge of the asymptotic structure of the wave function is required. They can be formulated independent from any boundary conditions. This is advantageous in those cases, where asymptotic behaviour at large interparticle distance is unknown or only known approximately, e.g. in scattering processes involving multi-particle continua.

In time-independent scattering e.g. electron impact ionization or in perturbative treatment of laser atom interactions, $L^2$ functions are commonly used. A prominent example are hydrogen-like Sturmian functions, which are used in the convergent close-coupling- and J-Matrix method^24–28^.

A Sturmian function approach to solve the TDSE was also employed by Hamido et al.^29^ and Frapiccini et al.^30^. The focus of their work is to identify time integration methods to avoid the problems associated with the stiffness of large equation systems. They present the total wave function as superpositions of Sturmian functions with time-dependent expansion coefficients and calculate multiphoton ionization of atomic H in laser fields. In these works, asymptotic boundary conditions are not imposed explicitly: The function space is chosen to be so big that numerical effects on the boundary of the $L^2$ function space play no role and the boundary is not overstepped.

Boundary conditions employed in the spatial domain can not be applied directly to the function domain, they differ substantially: In the $L^2$ approach the function space is divided into an inner part, spanned by $\{\phi _n^l\}_{n=0}^N$, and an outer part, spanned by $\{\phi _n^l\}_{n=N+1}^\infty$. Herein *l* is an integer and associated with the l-th partial wave. In the outer part, short range interactions are neglected and asymptotic solutions in the space $\{\phi _n^l\}_{n=N+1}^\infty$ are known analytically. Boundary conditions are imposed on the complex valued expansion coefficient $a_{\!N}^{\,l}$ of the function $\phi _{\!N}^{\,l}$ at the border of the function space. The function $\phi _{\!N}^{\,l}$ is delocalized in the radial domain and is not associated with a well defined radial value $r=R$ in the radial space. In the following part we use the term boundary condition in the $L^2$ function sense: An algebraic condition for the expansion coefficient at the border $n=N$ in the $L^2$ space to connect the inner part of the $L^2$ solution to the asymptotic part.

In the case of photoionization with emission of one electron or electron scattering with excitation, the asymptotic behaviour at large distances is governed by a central Coulomb force and solutions are known analytically, both in the position space and the function space. There outgoing waves $e^{ikr}$ with particle impulse *k* and position variable *r* are present. Then it is advantageous, to make use of the asymptotic $L^2$ solutions and to incorporate these directly in the boundary conditions. Because of the norm conserving character of the TDSE, in time propagation within a finite dimensional function space - and of course in position space with finite radii - there are always reflections at the numerical boundary, which result in unphysical values of the complex expansion coefficients. Then it would be desirable to make the boundary for these outgoing parts permeable and to absorb the reflected parts with the behaviour of $e^{-ikr}$. Such an approach is pursued in this work: Using a time step propagation scheme, the $L^2$ boundary is made permeable for at least one *k*-value of the impinging wave at each time step. Hadley^31^ applied such an approach in the spatial domain in the context of classical electrodynamics to calculate the dispersal of electromagnetic waves in beam propagation. In the spatial domain the concept of permeability is also employed by Givoli and Neta^32^, who introduce a multiplicative form of Sommerfeld’s differential equation for outgoing waves, which has to be fulfilled by the solution at the boundary. This ensures permeability for a theoretically randomly large number of discrete *k*-values. In practical calculations the maximum number of *k*-values has been limited by 3 and to the one-dimensional case.

In this article, we will adopt the concept of permeability for incident waves at the numerical boundary. In contrast to the works mentioned above, we formulate appropriate outgoing wave boundary conditions not in the position space but at the border of the $L^2$ function space. These time-dependent $L^2$ boundary conditions are based on the analytically known asymptotic solutions of the J-Matrix scattering theory and ensure permeability for a specified *k*-value for each time step and each partial wave. This way, at each time step the boundary can be made permeable for several different *k*-values. The flexibility of the method is further enhanced by adjusting the variable *k* in every time step. As such, the method is similar to the time-independent J-Matrix approach, whereby an expansion of the wave function in Sturmian functions is adjusted at the border of the $L^2$ function space to fit the expansion coefficients of the inner domain to those of the outer domain. The objectives of this article is three fold:

- Establishing an equation system for the one electron TDSE based on the mathematical foundations of the J- Matrix scattering theory and incorporating time-dependent asymptotic boundary conditions.
- Application to the photoionization of H in attosecond fields. This includes the numerical implementation and as a proof of the method comparing the results with values of other independent approaches. For this the H atom is exposed to few and half cycle laser fields.
- To assess how well Sturmian functions can describe the ionization dynamics in exterior fields whose strength is comparable with that of interior atomic fields.

To this purpose, we will first recapitulate the connection between Sturmian functions and Coulomb functions and then set up an equation system for the expansion coefficients. Subsequently, we derive the boundary conditions in the $L^2$ function space. Then we explicate the numerical realization and present the results. We first examine the interaction of H with a weak few cycle attosecond pulse and then the strong field half cycle case. Next the ionization dynamics in half cycle pulses is discussed and then the interaction of H with two delayed attosecond pulses is illustrated. Finally we mention possible applications for future work.

In this article the atomic units used are ( $e= \hslash = m_e = 1$).

## Theoretical methods

In time-independent collision theory, it was demonstrated in a series of papers^33–36^ how Sturmian functions can be efficiently used not only to describe the bound state part but also the continuum part of the collision system. It is therefore opportune, to transfer the concept of Sturmian functions so successfully used in time-independent scattering methods to time-dependent processes involving ionization. For example in the J-Matrix theory the $L^2$ function space is divided in an interior and exterior asymptotic part. Coulomb-Sturmians are used to present both parts on an equal footing, while at the boundary between the two parts the wave function is adjusted by the $L^2$ analogon of the R-Matrix^28^.

To begin with, Sturmian functions - which are the basis of the J-Matrix method - will be presented in short and then the algebraic description of the Schrödinger equation will be developed. This results in a system of differential equations of first order in time, which will serve as the basis for specifying boundary conditions and the numerical implementation.

### System of equations for the time propagation

The three-dimensional TDSE for an atom with core charge z is:

1 $$H\;\Psi (\vec{r},t)\; = \;(H_{0} + V)\;\Psi (\vec{r},t)\; = \;( - \frac{1}{2}\vec{\nabla }_{r} ^{2} \; - \;\frac{z}{r}\; + \;V(\vec{r},t)~)\;\Psi (\vec{r},t)\; = \;i{\mkern 1mu} \frac{\partial }{{\partial t}}\Psi (\vec{r},t)$$

Herein $V( \vec{r},t)$ is an exterior electric field in dipole approximation, either given in length, velocity or acceleration representation.

2a $$\begin{aligned}&V_L\,(\vec{r},t)&= \; \;\vec {E}(t) \,\cdot \, \vec{r} \end{aligned}$$

2b $$V_{V} {\mkern 1mu} (\vec{r},t) = \;\; - i\;\vec{A}(t){\mkern 1mu} \cdot {\mkern 1mu} \vec{\nabla }$$

2c $$V_{A} {\mkern 1mu} ({\vec{r}},t) = \;\;z~{\mkern 1mu} \alpha (t)\;{\mathbf{}}{\mkern 1mu} \vec{\epsilon}
\cdot {\mkern 1mu} \frac{{\vec{r}}}{{r^{3} }}\;,\quad \quad \quad {\vec{\alpha }}(t) = \int_{0}^{t} {\vec{A}}(t^{\prime})\;dt^{\prime}\;,\quad \quad \quad {\vec{A}}(t) = - \int_{0}^{t} {\vec{E}}(t^{\prime})\;dt^{\prime}$$

Herein $\vec {E}$ symbolizes the electric field strength, $\vec {A}$ the vector potential and the polarization $\vec {\epsilon }$ of the electric pulse is given through $\vec {\epsilon } = \vec {E}/\ E$,   $\vec {\alpha }$ is the excursion amplitude of the electron experienced by the field $\vec {E}$.

For (1) a partial wave decomposition is carried out and the wave function is expanded in the radial part in Coulomb-Sturmian functions $\phi _n^{l}(\xi \,r)$ and spherical harmonics $Y_{lm}$ in the angular part:

3 $$\begin{aligned} \psi \,(\vec {r},t) \;= \; \sum _{n=0}^{N}\,\sum _{l\,m} a_n^{lm}(t)\; Y_{lm} (\hat{r}) \; \frac{\phi _n^l(\xi r)}{r} \end{aligned}$$

The expansion is cutoff at an upper value *N* which characterizes the $L^2$ boundary. The time dependence is contained in the expansion coefficients $a_n^{lm}(t)$; the Sturmian functions are defined as follows:^34–36^

4 $$\phi _{n}^{l} (\xi {\mkern 1mu} r)\; = \;e^{{ - \xi r/2}} \;(\xi {\mkern 1mu} r)^{{l + 1}} \;L_{n}^{{2l + 1}} (\xi {\mkern 1mu} r)$$

These are regular at the origin and consist of Laguerre polynomials $L_n^{2l+1}$, powers in *r* and decaying exponentials. $\xi$ - within certain limits - is a freely selectable scaling parameter in radial direction. The function’s maximum is found to be roughly at $\frac{2n}{\xi }$. As a result, by varying $\xi$, various areas in position space can be sampled.

The functions $\phi ^l_n$ form a complete set, are not orthogonal, but tridiagonal, whereby $T^l_{n\,n'}{=}\int dr \, \phi ^l_n \, \phi ^l_{n'}$ only differs from 0 when $n'=n{-}1,n,n{+}1$. The integrals can be analytically evaluated: $T^l_{n\,n+1}=T^l_{n+1\,n} =-(n+1)_{2l+2}/\xi$ and $T^l_{n\,n} = 2(n+l+1)(n+1)_{2l+1}/\xi$, with the Pochhammer symbol $(a)_b= \Gamma (a+b)/\Gamma (a)$. The tridiagonality of the matrix elements also applies to the radial Hamilton operator with angular momentum *l*:^36^

5a $$\begin{aligned}&\int _0^{\infty }\,\phi _n^l\,\left( -\frac{1}{2}\frac{d^2}{d r^2}+\frac{l(l+1)}{2 r^2}-\frac{z}{r}\right) \phi _{n'}^l \;= \;h_{nn'}^l \end{aligned}$$

5b $$\begin{aligned}&h_{n\,n+1}^l = h_{n+1\,n}^l = \frac{\xi }{8}(n+1)_{2l+2}\;\; \; \;\; \; \;\; \; \text {and } \; \; h_{n\,n}^l= \left( \frac{\xi }{4}(n+l+1)-z\right) \, (n+1)_{2l+1} \end{aligned}$$

The expansion coefficients $a^{lm}_n$ will be determined using the Galerkin method on the one electron basis $\left\langle Y_{lm} \phi ^l_{n}\right|$, whereby *n* lies in the interval [0, *N*]. Projection on this basis leads to a coupled system of differential equations of first order in $a_{n}^{lm}$, thereby minimizing the deviation in the weighted mean for the expansion-coefficients:

6 $$\begin{aligned} \begin{aligned} \left\langle Y_{lm}\,\frac{\phi _n^l}{r} \, \big \vert \,H_0+V \,\big \vert \,\Psi (\vec {r},t)\right\rangle&= \left\langle Y_{lm}\,\frac{\phi _n^l}{r} \,\big \vert \,H_0+V\, \big \vert \sum _{l'm'}\sum _{n'=0}^N \, a_{n'}^{l'm'} \, Y_{l'm'}\frac{\phi _{n'}^{l'}}{r}\right\rangle \\&= ~~~~~ \,i \frac{\partial }{\partial t} \, \left( T_{n\,n-1}^{l}\,a_{n-1}^{lm} \,+ \,T_{n\,n}^{l}\,a_{n}^{lm} \, + \, T_{n\,n+1}^{l}\,a_{n+1}^{lm}\right) \end{aligned} \end{aligned}$$

Special attention has to be paid for the boundary *N*, which should be high enough to enclose the major part of the interaction term $V(\vec {r},t)$. For $n \ge N$ it is assumed, that the net photon absorption tends to $\sim 0$, meaning that the electric field can be neglected. In addition there the time derivative of $a_{N+1}^{lm}$ is disregarded, leading to the following approximation at the boundary $n=N$:

7 $$\begin{aligned} \left\langle Y_{lm}\,\frac{\phi _N^l}{r} \, \big \vert \, H_0+V \, \big \vert \,\Psi (\vec {r},t)\right\rangle \; = \; \,i \frac{\partial }{\partial t} \, \left( T_{N\,N-1}^{l}\,a_{N-1}^{lm} \,+ \,T_{N\,N}^{l}\,a_{N}^{lm}\right) \end{aligned}$$

Solving this equation according to $i \frac{\partial }{\partial \,t}\;a_N^{lm}$ gives:

8 $$\begin{aligned} i \frac{\partial }{\partial t}\,a_N^{lm}\;=\; \frac{1}{T_{N\,N}^l} \left\langle Y_{lm}\,\frac{\phi _N^l}{r} \, \big \vert \,H_0+V \,\big \vert \,\Psi (\vec {r},t)\right\rangle \, - \,i \, \frac{T_{N\,N-1}^l}{T_{N\,N}^l}\,\frac{\partial }{\partial \,t} \; a_{N-1}^{lm} \end{aligned}$$

Insertion into (6) with index $N-1$ leads to:

9 $$\begin{aligned} \begin{aligned} \left\langle Y_{lm} \,\frac{\phi _{N-1}^l}{r} \, \big \vert \,H_0 +V \, \big \vert \,\Psi \right\rangle -\frac{T_{N\,N-1}^l}{T_{N\,N}^l}\left\langle Y_{l\,m}\,\frac{\phi _N^l}{r} \, \big \vert \,H_0+V \, \big \vert \,\Psi \right\rangle = \quad \quad \quad \quad \quad \quad \quad \quad \quad \\ \;i \frac{\partial }{\partial \,t} \left[ T_{N-1\,N-2}^{l}\,a_{N-2}^{lm}+\left( T_{N-1\,N-1}^{l}-\frac{(T_{N\,N-1}^l)^2}{T_{N\,N}^{l}}\right) \,a_{N-1}^{lm}\right] \end{aligned} \end{aligned}$$

The matrix elements $T_{n\,n'}^l$ are time-independent, equation (6) with radial indices $n \in [0,N-2]$ together with (9) with $n=N-1$ defines *N* equations for the $N+1$ unknown coefficients $a_n^{lm}$ for each partial wave. The Galerkin method is independent of boundary conditions, up to now, no boundary conditions had entered into equations (6) and (9). A single degree of freedom remains, which can be shaped so as to create outgoing wave conditions at the boundary limit $n=N$. This condition and the calculation of $a_N^{lm}$ will be derived below.

### Boundary conditions

If the potential term V in (1) is ignored, the solutions are given by Coulomb functions with energy $E=k^2 / 2$. These exhibit the asymptotic behaviour $e^{+i( kr + \frac{1}{k} ln \; 2kr + \; l \frac{\pi }{2})}$ and $e^{-i( kr + \frac{1}{k} ln \; 2kr + \; l \frac{\pi }{2})}$ and form a fundamental system of $H_0$.

The corresponding Coulomb solutions $\Psi _l^+$ and $\Psi _l^-$ can be expanded in the infinite dimensional Sturmian basis $\phi _n^l$ and form the foundation of the J-Matrix method:^28,34–36^

10a $$\begin{aligned} \Psi _l^+ (r,k) \; = \;&\sum _{n=0}^{\infty } Q_n^{+l}(k,\xi ) \;\, \phi _n^l(\xi r) \end{aligned}$$

10b $$\begin{aligned} Q_n^{+l}(k,\xi )\; = \;&\frac{\xi }{2\pi \left( E+ \frac{\xi ^2}{8}\right) \, (n+1)_{2l+1}\;(2l+1)! \; \, \Psi _0^{+l}} \; q_n^{+l} \end{aligned}$$

The expansion coefficients $Q_n^{+l}$ consist of the Coulomb spectral-function $\Psi _0^{+l}$ and the Pollaczek functions $q_n^{+l}$, defined by

10c $$\begin{aligned} q_n^{+l}(k,\xi ) \; = \;&\frac{-2\, (n{+}2l{+}1)!\;\,(-\xi )^{n+1}\; \, \Gamma (l{+}1{-}\frac{iz}{k})}{\Gamma (n{+}l{+}2{-}\frac{iz}{k})} \; \; \, {}_2F_1\left( {-}l{-}\frac{iz}{k}; \, n{+}1; \, n{+}l{+}2{-}\frac{iz}{k}; \, \chi ^2\right) \end{aligned}$$

10d $$\begin{aligned} \Psi _0^{+l}\;= \;&\frac{e^{ \pi \frac{ z}{2k}} \; \; \; \Gamma ( l+1-\frac{iz}{k}) }{\sqrt{2\pi k} \; \;(2l+1)! } \; ( 2 \sin \gamma )^{l+1} \end{aligned}$$

and $\gamma$, $\chi$ and *x* are defined by

$$\begin{aligned} \sin \gamma = \sqrt{1-x^2} = \frac{k\xi /2}{E+\xi ^2/8}, \;\;\;\;\; \; \; \chi = \frac{\xi +2ik}{\xi -2ik} = e^{i\gamma }, \;\;\;\;\; \; \; x = \frac{E-\xi ^2/8}{E+\xi ^2/8} = -\cos \gamma \end{aligned}$$

The second solution of the fundamental system $(\Psi _l^+, \Psi _l^- )$ arises via substitution $k\rightarrow -k$. It needs to be mentioned that functions $\Psi _l^+$ and $\Psi _l^-$ only merge with corresponding Coulomb functions in the asymptotic limit $r \rightarrow \infty$ when *n*-values are large, because with the $L^2$ basis $\phi _n^l$ the irregular behaviour $\sim 1/r^{l+1}$ at the origin cannot be reconstructed.

The various forms of the dipole operator show different behaviours for $r\rightarrow \infty$. The acceleration operator scales like $1/r^2$ for $r\rightarrow \infty$, whereas the length and velocity form grow unhindered. In order to nevertheless formulate boundary conditions in the asymptotic function space for these cases, it is hypothesized that for large *n*-values the effect of the dipole operator can be neglected - this is equivalent that absorption and emission of photons occurs only in a restricted area around the nucleus. This approximation is strictly true only for the acceleration form, where only a restricted domain around $r=0$ contributes in the interaction, but is also adopted here for length and velocity representation. With this approximation and assuming an unlimited propagation space, the coefficients $a_n^{lm}$ in (3) have to show outgoing wave behaviour for large *n* and each partial wave and are thus proportional to the Pollaczek-functions $Q_n^{+l}$ appearing in (10a):

11 $$\begin{aligned} a_{n}^{lm}(t) \ \sim \ Q_n^{+l} \ \ \text {for} \ \ n \rightarrow \infty \end{aligned}$$

For sufficient large indices *n* the time evolution is entirely governed by the free field operator $H_0$ and thus the quotient of the expansion coefficients $a_{n-1}^{lm} / a_n^{lm}$ in consecutive $n-1, \, n$ can be regarded as approximately equal. Then the following two equations at the boundary can be set up for the quotient of two consecutive coefficients:

12a $$\begin{aligned} \frac{a_{N-2}^{lm}(t)}{a_{N-1}^{lm}(t)} \; = \;&\frac{Q_{N-2}^{+l}\,(k,\xi )}{Q_{N-1}^{+l}\,(k,\xi )} \end{aligned}$$

12b $$\begin{aligned} \frac{a_{N-1}^{lm}(t)}{a_{N}^{lm}(t)} \; = \;&\frac{Q_{N-1}^{+l}\,(k,\xi )}{Q_{N}^{+l}\,(k,\xi )} \end{aligned}$$

At each time point $t_i$ in the time propagation the values of $a_{n}^{lm}$ for $n \in [0, N-1]$ are known, especially the values of $a_{N-2}^{lm}$ and $a_{N-1}^{lm}$. It is then possible to solve equation (12a) for a complex *k*:

13 $$\begin{aligned} \frac{a_{N-2}^{lm}(t)}{a_{N-1}^{lm}(t)} \;- \; \frac{N+2l}{N-1}\;\frac{q_{N-2}^{+l}\,(k,\xi )}{q_{N-1}^{+l}\,(k,\xi )} \; = \; 0 \end{aligned}$$

Herein, for pure outgoing behaviour, *k* is real and $>0$. Due to reflections at the numerical boundary, *k* can take on complex values: The case $Re(k) < 0$ corresponds to the unphysical event where the impinging wave is scattered back. So the procedure to avoid reflections and guaranteeing outgoing waves consists of setting $Re(k) =0$ when $Re(k) < 0$ in each time step and each partial wave. With the *k*-value thus determined and adjusted if $Re(k) < 0$, the expansion coefficient $a^{lm}_N$ can then be computed via the equation (12b):

14 $$\begin{aligned} a_{N}^{lm}(t) \; = \; \frac{N}{N+2l+1}\;\frac{q_{N}^{+l}\,(k,\xi )}{q_{N-1}^{+l}\,(k,\xi )}\;a_{N-1}^{lm}(t) \end{aligned}$$

In the high energy limit $k\rightarrow \infty$ the Pollaczek-function can be analytically evaluated. This yields the limit condition:

15 $$\begin{aligned} a_{N}^{lm}(t)\; = \; \frac{N}{N+2l+1}\;a_{N-1}^{lm} \qquad \qquad k\longrightarrow \infty \end{aligned}$$

Equations (13) and (14) are solved at each time step and for each partial wave. Thus *k* can vary and can be adjusted in each time step for each *l*. Using this procedure the probability of the electron to be in the interior zone $n < N$ of the $L^2$ function space decreases. This is also evident in the numerical simulations, where the test cases show a decreasing probability, dependent on the basis size and the scaling parameter involved.

### Role of gauge

All three forms of the transitional operator (2) are equivalent given an infinite dimensional function space ($N {\rightarrow } \infty$) and are connected via a gauge transformation. Because the operators are vectors, they only connect angular momenta differing by $\pm 1$ and states of different parity. Differences occur in their action in configuration and function space: The acceleration form corresponds to the Kramer-Henneberger’s reference system and results when a Taylor expansion of $\frac{1}{|\vec{r}+\vec{\alpha} |}$ for a small quiver amplitude $\alpha$ is taken. Thereby regions close to the core are sampled and for $r \rightarrow \infty$ the asymptotic behaviour is characterized by $\frac{1}{r^2}$. In contrast, the length and velocity forms grow without limit for $r \rightarrow \infty$.

Like the matrix elements for $H_0$, the elements for the length and velocity form of the dipole operator show a narrow banded structure, while those for the acceleration form are not banded. The operators $V_L$ and $V_V$ connect only adjacent indices *n*, while $V_A$ leads to transitions to all Sturmians with $n' \ge n$ :

16a $$\begin{aligned} \left\langle Y_{l m}\frac{\phi _n^{l}}{r} \big \vert \ V_L \ \big \vert Y_{l' m'}\frac{\phi _{n'}^{l'}}{r} \right\rangle \ne 0 \qquad \qquad&\text { for} \ l'=l{+}1, \qquad n' \in [n{-}3 \; , \;n{+}1] \nonumber \\&\text { or} \ \ l'=l{-}1, \qquad n' \in [n{-}1 \; , \; n{+}3] \end{aligned}$$

16b $$\begin{aligned} \left\langle Y_{l m}\frac{\phi _n^{l}}{r} \big \vert \ V_V \ \big \vert Y_{l' m'}\frac{\phi _{n'}^{l'}}{r} \right\rangle \ne 0 \qquad \qquad&\text { for} \ l'=l{+}1, \qquad n' =n{-}2 \; \; or \; \; n' = n \nonumber \\&\text { or} \ \ l'=l{-}1, \qquad n' =n+2 \; \; or \; \; n'=n \end{aligned}$$

16c $$\begin{aligned} \left\langle Y_{l m}\frac{\phi _n^{l}}{r} \big \vert \ V_A \ \big \vert Y_{l' m'}\frac{\phi _{n'}^{l'}}{r} \right\rangle \ne 0 \qquad \qquad&\text { for} \ l'=l{+}1, \qquad n' \ge n \nonumber \\&\text { or} \ \ l'=l{-}1, \qquad n' \le n \end{aligned}$$

With the length form, only orbitals with $n' \in [ -3, +3]$ can be reached. Per time step the wave function spreads outward by three $\phi _n^l$ units. With the velocity form, the wave function propagates in time by two functions in the $L^2$ function space.

Despite the fact that in position space the acceleration form is well localized at the origin, it spreads much more rapidly in the Sturmian function space. The behaviour is determined via the matrix element $\int _0^\infty dr \phi _n^l\ \frac{1}{r^2} \;\phi _{n'}^{l+1}\;$. This is $\ne 0$ for all $n' \ge n$, i.e. in time propagation the whole $\phi _n^l$ space will already be occupied in the first time step, although it will decrease as $n'$-values rise. The acceleration form is nonlocal in the indices $n,n'$ and therefore less suited for time propagation. Evidence for this is also supported by numerical tests: In order to achieve convergence for the acceleration form, *N* has to be chosen much larger than in the length or velocity case with enormous costs on computer ressources.

Summing up: The length form and the velocity form are suitable for time propagation and provide comparable results to circa three digits. The acceleration form, however, is less suitable for the numerical time propagation within a Sturmian function approach.

### Observables

After turning off the external field, relaxation occurs and the system can be described as a superposition of Coulomb waves. All information about ionization processes is contained in the expansion coefficients $a_n^{lm}(t)$. Physical observables like energy spectra are obtained by projecting $\Psi (\mathbf {r},T_f)$ on Coulomb functions $\Psi _{\mathbf {k}}^-$. These are normalized to incoming wave boundary conditions and correspond to an ionization experiment in which the energy of the photoelectron is measured after the ionization process.

17 $$\begin{aligned} \Psi (\vec {r},T_f) = \int k^2\; dk\; d\Omega _k\; b_{\vec {k}}\;\Psi _{\vec {k}}^- \end{aligned}$$

The Coulomb wave $\Psi _{\vec {k}}^-$ can be expanded in spherical harmonics in the impulse and position directions and an energy-dependent radial function $\Psi _E^L$.

18 $$\begin{aligned} \Psi _{\vec {k}}^- \; = \; \frac{1}{kr} \,\sum _{lm}\,i^l \; Y_{lm}^* (\hat{k})\;Y_{lm}(\hat{r})\;e^{-i\sigma _l}\; \Psi _E^l \end{aligned}$$

Herein $\sigma _l (k)$ is the Coulomb scattering phase, given as $arg\,[\Gamma (l+1-i\;\frac{z}{k})]$, and $\Psi _E^l$ corresponds up to a phase factor to the regular Coulomb function, normalized to an energy delta function.^37^

19 $$\begin{aligned} \Psi _E^l \;= \; e^{\pi \frac{z}{2k}}\; \frac{|\Gamma (l\!+ \! 1 \!-\!i \frac{z}{k})|}{\sqrt{2\pi k} \;(2l+1)!}\;(2kr)^{l+1}\;e^{ikr}\; \Phi \,(l\!+\!1\!-\!i\;\frac{z}{k},\,2l\!+\!2,\,-2ikr) \end{aligned}$$

$\Psi _E^l$ can, as is shown in the J-Matrix theory, be expanded according to the $L^2$ functions $\phi _n^l$ :^36^

20 $$\begin{aligned} \Psi _E^l(k,r) \; = \; \left| \Psi _0^{+l}\right| \;\sum _{n=0}^{\infty }\; \frac{n! \; (2l+1)!}{(n+2l+1)!}\;p_n^l(x,\xi )\; \,\phi _n^l(\xi r) \end{aligned}$$

with $\Psi _0^{+l}$ and x given by (10) and $p_n^l$ is a Pollaczek-polynomial^36^. The function $b_{\vec{k}}$ is a measure how much a Coulomb wave with energy *E* and impuls $\vec {k}$ contributes to the solution $\Psi (\vec {r}, T_f)$. The impulse distribution of the photoionized electrons can be further separated into an energy part $c_E^{lm}$ with $E=k^2/2$ and an angle part. The angle part of the electron impulse is characterized by spherical harmonics and expanded as following:

21 $$\begin{aligned} b_{\vec{k}}\; = \; \sum _{l\;m} c_E^{lm} \;Y_{lm}(\hat{k}) \end{aligned}$$

Insertion in (17) results in the following representation:

22 $$\begin{aligned} \Psi (\vec{r},T_f) \; = \; \sum _{lm}\;\sum _{l'm'}\; \int k^2 dk \; d\Omega \; c_E^{lm} \; Y_{lm}(\hat{k}) \; \frac{1}{kr} \; i^{l'} \; Y_{l'm'}^*(\hat{k}) \; Y_{l'm'}(\hat{r})\; e^{-i\sigma _l'}\; \Psi _E^{l'}(k,r) \end{aligned}$$

Integration over the angle part $d\Omega _k$ and projection with $\langle Y_{lm}\;\Psi _E^l |$ gives:

23 $$\begin{aligned} c_E^{lm} \; = \; (-i)^l\,e^{i\sigma _l} \left\langle Y_{lm}(\hat{r})\;\Psi _E^l(r)\;|\;\Psi ( \vec {r},T_f)\right\rangle \end{aligned}$$

The angle integrated cross section *dP*/*dE*, differential in energy, is defined as:

24 $$\begin{aligned} \frac{dP}{dE} \; = \; \sum _{lm} c_E^{lm} \; c_E^{lm \;*} \end{aligned}$$

The coefficients $c_E^{lm}$ can be determined analytically using the tridiagonal structure of the matrix elements of $\phi _n^l$. The result is

25 $$\begin{aligned} c_E^{lm} \; = \; (-i)^l\,e^{i\sigma _l}\;\left| \Psi _0^{+l}\right| \;\frac{\xi }{2}\;\frac{1}{E+\frac{\xi ^2}{8}}\;\sum _{n=0}^N\;\left( n+l+1-\frac{2z}{\xi }\right) \;a_n^{lm} (T_f)\; \, p_n^l(E,\xi ) \end{aligned}$$

## Numerical realization

The equation system (6) and (9), together with the boundary condition (13) and (14), represents a differential equation system of first order for the expansion coefficients $a_n^{lm}$, which is to be solved numerically by a Runge-Kutta procedure. Written in vectorial notation:

26a $$\begin{aligned} H(t) \, a(t) \; = \; i\frac{d}{dt} T ~ a(t) \end{aligned}$$

The matrix T is tridiagonal, time-independent and consists of the coefficients from the right side of (6) and (7). A transformation $y(t) = T a (t)$ leads to the standard form:

26b $$\begin{aligned} H(t) \; T^{-1} \, y(t) \; = \; i \frac{d}{dt} ~ y(t) \end{aligned}$$

The Hamilton matrix H in the basis of $\phi _n^l$ is block diagonal in *l*, the Hamilton matrix without laser interaction is tridiagonal and the dipole operator is pentadiagonal (depending on whether the length or velocity form is used). The time propagation is implemented with a Runge-Kutta method of $8^{th}$ order with constant step width, depending on the dimension of the equation system. At each time step a matrix vector multiplication $T^{-1}y$ has to be carried out, and the resulting vector is then multiplied with the Hamilton Matrix. Due to the sparse occupation of H, only a few calculation steps are involved. In order to achieve outgoing waves at the limit of the $L^2$ function space it is necessary, at every time step, to determine the value $a_N^{lm}$ pursuant to (14). To this purpose, the complex value of *k* is determined in accordance with (13). The Pollaczek functions $q_n^{+l}$ are complex-valued and are calculated by means of the Gauss continued fraction. Due to the partial wave expansion, the corresponding *k*-values satisfying (12a) can be adjusted specifically for each *l*. So at each time step, the boundary can be made permeable for several *k*-values, thus enhancing the flexibilty of the boundary approach (11). The *k*-values are determined by a Newton procedure to determine a zero of (13). Because the *k*-values only change slowly from one time step to the next, the *k*-values of the previous step are used as a starting point for the Newton procedure, resulting in fast convergence after 3 - 4 steps. As can be seen in (12a) for determining the *k*-values, there is no coupling between different *l*. This makes it possible in the computer code to do the calculation in parallel on a multiprocessor system using a multithreading library.

The differential system (6) is stiff, resulting in decreasing time steps with increasing basis size. This is because the positive energy spectrum is not bounded. Increasing the basis size results in approximating more and more higher energy states, which oscillate strongly dependent on the energy. Thus for longer interaction times and huge basis sizes , a Runge-Kutta method is not the favourite method, in this case Lanczos procedures or a method proposed by Fatunla^30,38^ can be considered as alternatives. A further approch to overcome stiffness and to extend the method into the femtosecond range would be to transform to a spectral representation by diagonalizing a large Hamilton matrix and to disregard the high energy eigenvectors. In the simulations presented in the following part the time steps are adjusted properly and differ in the specific calculations dependent on the maximum number of functions used in the expansion (3).

All the calculations were carried out either in with the velocity or the length form of the dipole operator. The results using length or velocity form differ only minimally from one another and agree within the first 2 - 3 digits. This is shown in Table 1 where the angle-integrated ionization probabilities at different energy values of the continuum electron are presented; length form shown in $2^{nd}$ column, velocity form in $3^{rd}$ column. The calculation is based on an interaction with $E(t) = E_0 \, \sin (\omega t)$ with $E_0=1, \, \omega =2$ and a duration $T = \frac{2\pi }{\omega } = \pi$; it was calculated with 16 partial waves, each with 75 radial functions. For the results presented below a software package (Apfloat) was used for the numerical implementation. It permits calculations of arbitrary precision. Typically, 16- 25 digits were used. In order to achieve reliable, i.e. convergent results, the maximal number of partial waves, the number of radial functions *N* and also the scaling parameter $\xi$ were varied. The results shown in the following section can be considered to be convergent with respect to these parameters.

**Table 1:** Angle integrated ionization probabilities $d\!P/d\!E$ for specific energies, either calculated with length or velocity form of the dipole operator. Column 1: energy of the emitted electron, column 2: $d\!P/d\!E$ calculated in length form, column 3: in velocity form.   The elctrical pulse is specified through $E(t) = E_0 \; \sin (\omega t)$ , $E_0=1, \; \omega =2$ and a duration $T = \frac{2\pi }{\omega } = \pi$.

Energy	$d\!P/d\!E$ Length form	$d\!P/d\!E$ Velocity form
1.0 E-7	6.812795733 E-1	6.814726531 E-1
1.0	4.991422878 E-2	4.991538581 E-2
2.0	1.120858896 E-2	1.121393579 E-2
3.0	2.706846038 E-3	2.705325366 E-3
4.0	5.470143966 E-4	5.458633989 E-4
5.0	9.416780915 E-5	9.510984557 E-5
6.0	1.650235575 E-5	1.606840606 E-5
7.0	3.542347196 E-6	3.577486619 E-6
8.0	9.787512464 E-7	8.938820317 E-7

## Results and discussion

Apart from the stability of the computed values with regard to variation of the parameters like basis size, numerically predicted physical observables also have to be consistent with those of other work. This is pursued in this section where the quality of the $L^2$ expansion is demonstrated. First, two cases are examined:

1. In the weak field perturbative regime, the method is applied to a single attosecond pulse and results are compared with those of Della Picca^39^ *et al.* who use a differencing scheme on a spatial grid.
2. In the strong field case, the ionization of H in a half cycle pulse is investigated and compared with results of Duchateau et. al.^40^ who apply a B-Spline approach. The study is continued examining the dynamics of H in half cycle pulses and in a 12 cycle pulse. The results are compared with the First Magnus Approximation of Dimitrovski et al.^41^.

As a further application of the method, interference phenomena in two delayed attosecond pulses are analyzed.

### Comparison with B-spline and finite difference methods

To test the quality of the expansion in the perturbative regime, the same laser parameters used by Della Picca et. al.^39^ are chosen:

27 $$\begin{aligned} E(t) \; = \; E_0 \, sin[\omega ( t-t_0)] \; sin^2 \frac{\pi t}{T_f} ~,~~~\;\; t_0 = \frac{T_f-\pi }{2} ~, ~~~~ \; 0 \le t \le T_f \end{aligned}$$

**Figure 1:**
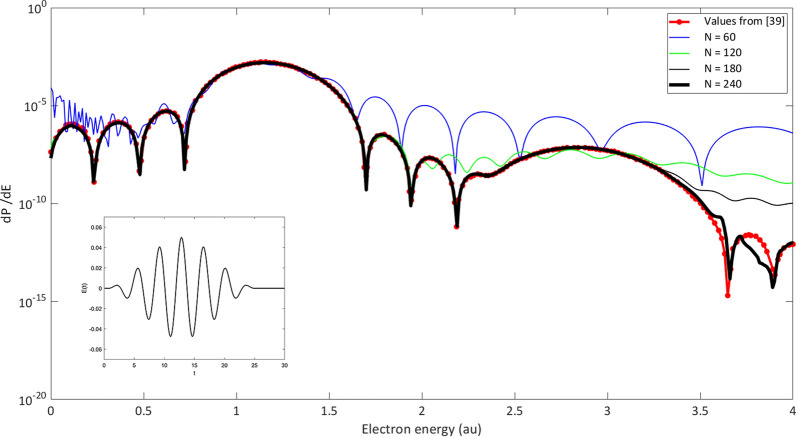
Ionization spektrum of H after irradiation with a linear polarized few cycle pulse. The electric field of the pulse is depicted in the inlay, the pulse parameters are $E(t) = E_0 \ sin[\omega ( t-t_0)] \; sin^2 \frac{\pi t}{T_f}$, $\omega = 1.71$, $T_f = 25.72$, $E_0 = 0.05$. Thick black solid curve (N=240): this work; red dotted curve: results from [39]. The thin curves in blue ($N=60$), green ($N=120$) and black ($N=180$) correspond to different values of the cutoff parameter N in (3).

Herein $\omega = 1.71$, the number of cycles = 7, the total duration $T_f = 25.72$, field strength $E_0 = 0.05$ with linear polarization in z-direction. Fig. 1 shows the ionization spectrum of H with the ground state as the initial state. For the calculation, the velocity form was used and the convergence with respect to variation of the scaling parameter $\xi$ and the basis size was tested. The electric field amplitude is considerably smaller than the inner atomic field strength. Thus, the one photon absorption dominates in the ionization spectrum, which can be clearly seen around $E=1.21$, where a maximum appears. At 2.9 a.u., the second peak turns out considerably smaller and corresponds to a two photon absorption. Due to the duration of 25.72 a.u., the laser pulse is of course not monochromatic, but spectrally distributed across the interval $\bigtriangleup \omega = \frac{4\pi }{T_f}\approx 0.5$. This also explains why the peaks at 1.21 and 2.91 are relatively wide. The significant structures in the area of the maximum at E= 1.2 are reproduced very well even with a basis size of 4 partial waves and 60 radial functions. In contrast, the structures in the area above 3.3 are smaller than at the maximum at $E =1.2$ by a factor $\sim$ $10^{-7}$. In order to resolve this area, it was necessary to adopt equations with eight partial waves and 240 radial functions in the $L^2$ expansion. To give an impression about the convergence behaviour, calculations with different basis sizes for the cutoff parameter *N* in the expansion (3) are included. In all calculations 8 partial waves were employed. For $N=60$ - thin green curve - the approximation is poor and only at the maximum at $E \sim 1.3$ the result is reliable. For higher $N \;(N=120, 180, 240)$ the convergence behaviour improves, especially in the higher energy part above 1.8.

**Figure 2:**
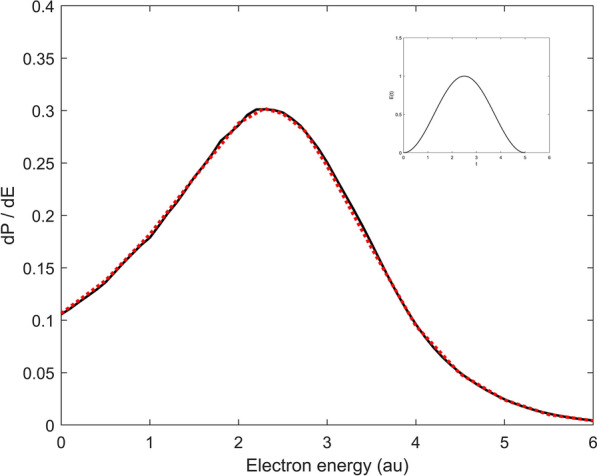
Ionization spektrum of H after irradiation with a half cycle pulse. The electric field of the half cycle pulse is depicted in the inlay, the pulse parameters are$E(t) = E_0 \; sin( \omega t \! - \! \omega T_f/ 2 \!+ \! \pi \! / 2) \; sin^2( \pi t / T_f), \, E_0 = 1, \, \omega = 0.05$ with total duration $T_f = 5.$Black solid curve: this work; red dotted curve: results from [40].

For comparison, results from [39] were integrated and shown by the red dotted solid curve . The minor differences above 3.5 a.u. can eventually be explained by the limited basis size and correspond to the high energy part of the wave packet which propagates outside in radial direction very quickly and thus reaches larger radial distances. The agreement - aside from the very weakly pronounced structure above 3.5 a.u - is remarkable and shows that a hydrogen like Sturmian basis can very well approximate the ionization dynamics - at least in the perturbative regime. It is, of course, of particular interest just how accurate the processes can be described when the strength of the exterior field is of the same magnitude as the inner atomic field strength. To this purpose, calculations were carried out with the H atom exposed for a half cycle to an intense field with amplitude $E_0 = 1$. To ensure comparability, the same field parameters as in Duchateau et.al.^40^ were taken: $E(t) = E_0 \, sin( \omega t - \omega T_f/ 2+ \pi / 2) \; sin^2( \pi t / T_f), \; E_0 = 1, \, \omega = 0.05$ with total duration $T_f = 5$. The time dependence of the electric field is illustrated in the inlet of Fig. 2; in contrast to Fig. 1 the ionization spectrum is depicted on a linear scale. Owing to the short pulse time, the spectral band width is significantly larger than in the previous example. The energy spread in the ionization spectrum is correspondingly wider. The values of Duchateau et. al.^40^ are added as red dots for purposes of comparison and display an excellent agreement. Duchateau et. al. used a B-spline basis for the expansion of the wave function and extracted the ionization spectrum via projection on Coulomb functions. The calculations in this work were carried out with 15 partial waves, each with 100 radial functions; the results are converged according to the basis size.

**Figure 3:**
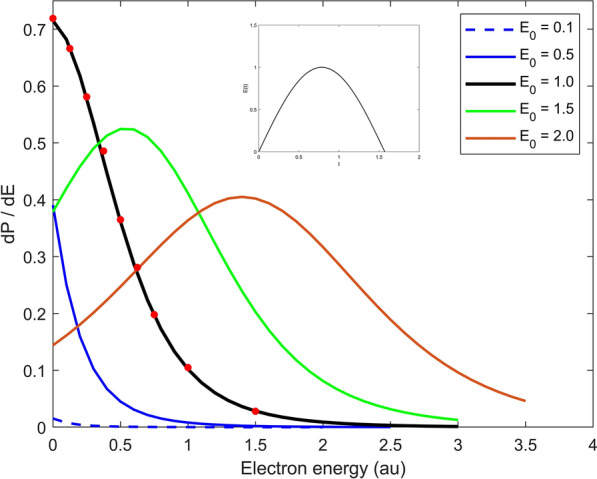
Ionization spektrum of H after irradiation with a half cycle pulse. The electric field of the half cycle pulse is depicted in the inlay: $E(t) = E_0 \; sin(\omega t), \,\, \omega =2$. The full curves correspond to different amplitudes of the half cycle pulse, the red dots are taken from Dimitrovski et al.^41^ and correspond to q=1 of the First Magnus Approximation.

**Figure 4:**
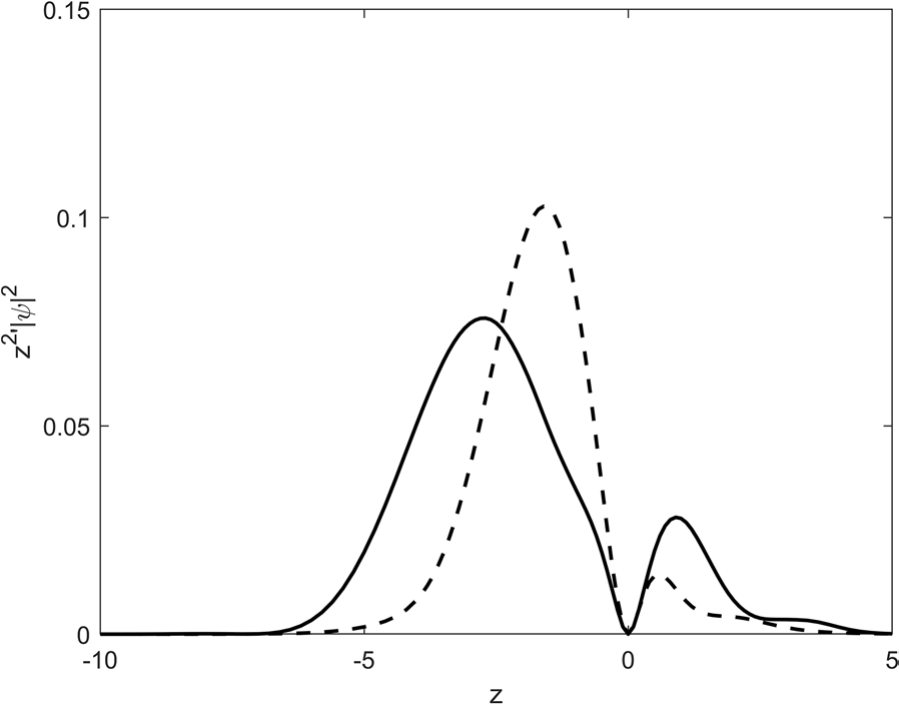
Electron density along the z-axis. Full curve: after passage of a half cycle pulse, dashed curve: after one full cycle Field parameters: $E(t) = E_0 \; sin(\omega t), \,\, \omega =2, \,\, E_0 = 1.0$

To sum up: From the time-independent J-Matrix method it is to be expected that an expansion in $L^2$ Sturmians, normally used for bound states, should be well suited to describe ionization processes in weak external fields - this is confirmed in Fig. 1. Furthermore, Fig. 2 gives convincing results that the $L^2$ expansion can also be applied to strong field processes in which the division of the system into ‘atom’ and ‘outer field’ no longer seems appropriate.

Building on these results, the following section examines the ionization dynamics of H in $0.5 , \; 1 , \; 1.5 \;$ and 12 cycle fields.

**Figure 5:**
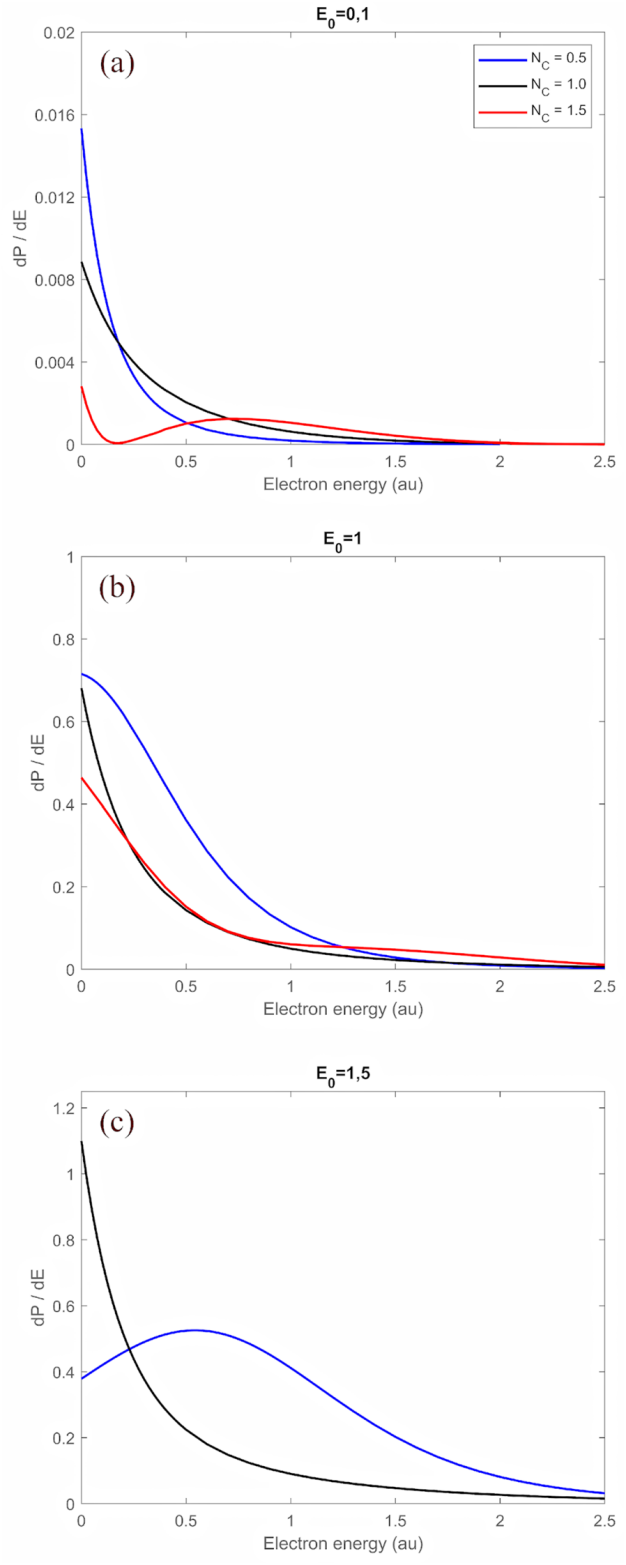
Ionization spectra of H for different numbers of half cycles $N_c$ and different field amplitudes $E_0$. (**a**): $E_0 = 0.1$, (**b**): $E_0 = 1.0$, (**c**): $E_0 = 1.5$. Please note, the diagrams contain different scalings of $d\!P/d\!E$.

**Figure 6:**
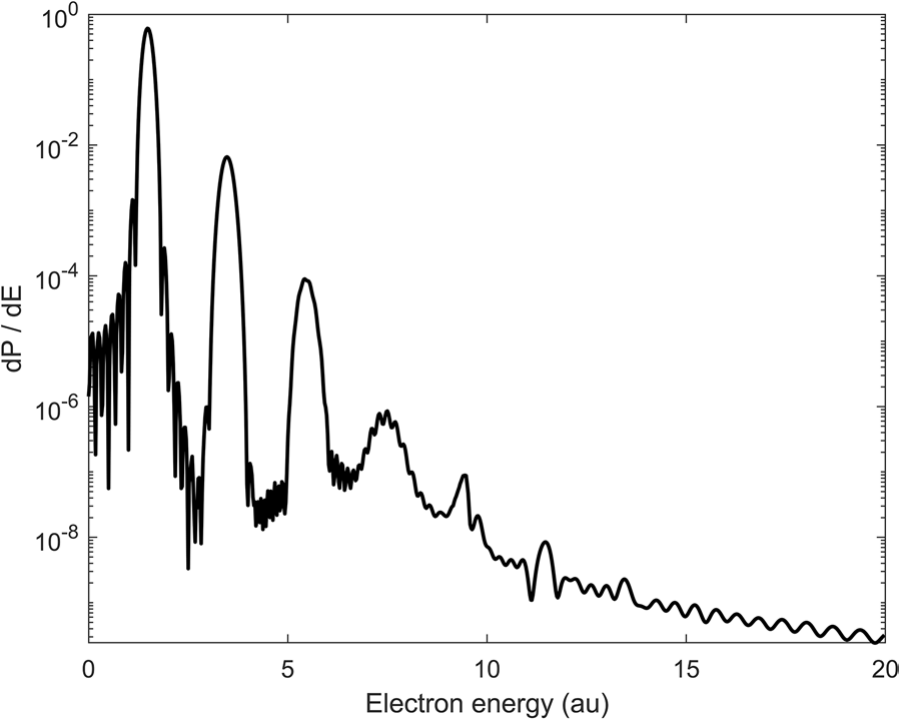
Ionization spectrum after passage of a 12 cycle pulse with field parameters: $E(t) = E_0 \; sin(\omega t), \,\, \omega =2, \,\, E_0 = 1.0$.

### Dynamics of H ionization in half and few cycle pulses

In this part, the effects of a half cycle pulse (HCP) on photoelectron spectra are considered. HCPs are a field of actual research, theoretically as well as experimentally^13,42–45^. A HCP is an idealization and does not exist in reality; the time integral over the electromagnetic signal has to be zero. A HCP can be approximated by an unipolar one cycle pulse where the electric field in the first half cycle ist strongly peaked while the second half cycle is strongly prolonged with a much lower amplitude.

**Figure 7:**
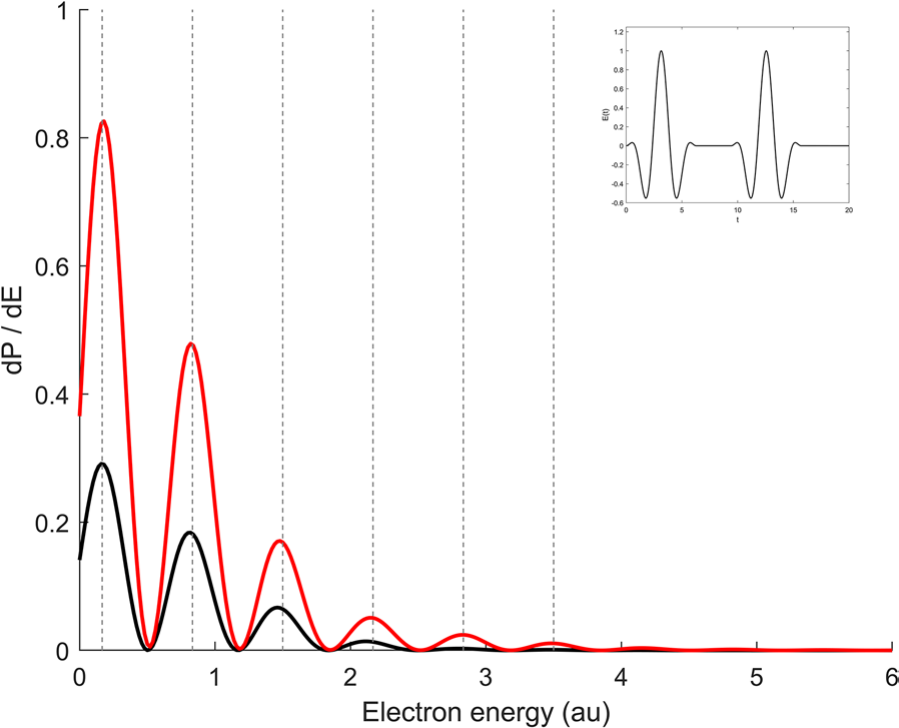
Ionization spektrum of H after irradiation with two delayed pulses according to (31). The electric field of the pulse is depicted in the inlay with $N_1 = N_2= 2, \, \omega =2, \, T_D=2\pi /\omega$. The curves correspond to different field amplitudes: black curve: $E_0=1$; red curve: $E_0=2$. The vertical lines illustrate the positions of the maxima predicted by (34).

To begin with, the H atom, initially in its ground state, is exposed to one HCP with linear polarization of the form:

28 $$\begin{aligned} E(t) = E_0 \; sin(\omega t) , \quad \quad \omega =2, \quad \quad t \in [0, T_f] \end{aligned}$$

Fig. 3 shows the ionization spectrum after passage of a HCP with duration $T_f = 0.5 \; \frac{2 \pi }{\omega }$ for different values of the electric field amplitude: $E_0 = 0.1, \; 0.5, \; 1.0, \; 1.5, \; 2.0$. These values comprise intensities which can be treated via perturbation theory, and values, which are comparable to, or larger than the inner atomic field strength. From Fig. 3 it becomes evident that for larger values of the field amplitude $E_0$ a significantly pronounced maximum appears that begins for $E_0 > 1$.

This behaviour stands in excellent congruence with the short time approximation, i.e. First Magnus Approximation, developed by Dimitrovski *et al.*^41^. In this approach, the transition probability for short interaction times (compared to the atomic orbital time of electrons) is expressed through:

29 $$\begin{aligned} P_{fi} = \left| \left\langle \phi _{f} \vert \; e^{-i \vec {q} \vec {r}} \; \vert \phi _{i} \right\rangle \right| ^2 \end{aligned}$$

The transition probability is independent of the exact form of the electric pulse and only dependent on the time integral over the elctric field $\vec {q} = \int _0^t dt' \vec {E}(t')$, which describes a momentum transfer averaged over time. The same matrix element also appears in atomic scattering processes in the first Born approximation. In this case, q designates the momentum transfer of the incident particle on the target atom. The final state wave function can be approximated for larger kinetic energies and, therefore, for large momentum transfers q, by plain waves. For the matrix element this results in^41^:

30 $$\begin{aligned} P_{fi} = \frac{(2z)^5}{4\pi ^2} \; \frac{1}{( |\vec {q}+\vec {k}|^2 +z^2)^4} \end{aligned}$$

It is this very behaviour that Fig. 3 illustrates, although here it is calculated by solving the three-dimensional Schrödinger equation with an $L^2$ approach. According to (30), the maximum ionization probability is $E = \frac{q^2}{2} - |E_i|$, where $E_i$ denotes the ground state energy of the H atom. For $q <1$ this maximum is in the negative energy range, which doesn’t contribute to the ionization spectrum. For $q > 1$ the maximum shifts further outward into the positive, and therefore measurable, energy range. The correspondence of the ‘exact’ solution of the TDSE, and the results as predicted by the First Magnus Approximation, is excellent. In Fig. 3, the black curve with field amplitude $E_0$=1 corresponds to the q = 1 case of the short time Magnus approximation, these are shown as red dots and are nearly congruent.

Figure 4 shows the electron density along the z-axis after passage of one HCP and two HCPs, each with linear polarization pointing along the z-direction and again with the H atom initially in its ground state. In the one HCP case the density is strongly located at negative *z*-values with a maximum at $z \sim 3.0$. When one observes a pulse consisting of a full oscillation cycle, the electric field in the second half cycle reverses its sign: the electron is slowed down and electrons with low energies are accelerated back toward the nucleus; there they can eventually recombine to bound states. This behaviour is clearly evident in the full cycle case shown by the dashed curve in Fig. 4. The electron density is shifted to smaller negative *z*-values. To sum up, the electron density shows, that, even after a symmetrical pulse (here, after a whole cycle) and a spatial isotropic initial state (here 1s ground state), the system is asymmetrically localized along the electric field direction.

In Fig. 5, the energy spectrum after interaction with one, two and three HCPs is presented, each for different field amplitudes $E_0$. In the $E_0=0.1$ case ( upper panel) and after passage of one HCP, electrons are mainly emitted with energies $\sim 0$ and the probabilty drops down during the second cycle, while for electron energies $> 0.3$ the probabilty increases slightly - see the black curve. The case $E_0=0.1$ corresponds to the perturbative regime, the electric field in the second half cycle is so weak, that it can only accelerate back the low energy electrons freed in the first half cycle. The situation changes with increasing field strength, as can be seen from the mid and bottom panel of Fig. 5. Here the field strength is ‘high enough’ in the second half cycle to effect also the higher energy electrons, emitted in the first half cycle. In the very strong field case with $E_0=1.5$ ( bottom panel) during the second half cycle the electron density increases around threshold and decreases for higher energies. High energy electrons freed in the first half cycle are decelerated and return back to the nucleus, but a considerable fraction has still positive energy, even after deceleration.

If the number of cycles is increased, the ionization spectrum shows the characteristic peak structure of above-threshold ionization corresponding to the long time limit: In Fig. 6, the ionization spectrum for an interaction time of 12 whole oscillation cycles with $\omega =2$ and $E_0=1$ is presented, this time on a logarithmic scale. The spectral width is reduced in comparison to one and two HCPs; the pulse is significantly more monochromatic. This effects the spectral energy distribution: Above the threshold peaks appear; their magnitudes decline with increasing order. They correspond to absorption of photons of frequency $\omega =2$: The first at $\sim 1.5$ and the other at energies $E = n \omega - E_i$, where $E_i$ is the ground state energy and n specifies the number of photons absorbed.

Summarizing the results from Fig. 3, 4, 5 and 6:

- The ionization spectrum in the one half cycle case shows excellent agreement with the First Magnus Approximation calculation of Dimitrovski et. al.^41^.
- In the case of one whole interaction cycle, recombination occurs in the second half wave, which leads to reduced ionization, but the ionization outweighs the recombination.
- Increasing the number of field cycles, the charcteristic above-threshold ionization structure - with peaks for integral multiples of the oscillation frequency - are successfully reproduced.

The methods developed in this work can be extended to more complex tailored fields, for example delayed attosecond pulses. An example of this will be examined below.

### Delayed attosecond trains

In the case of several time-delayed attosecond pulses, interference patterns in the ionization spectrum occur, which scale with the time delay between the individual pulses. In this part, the ionization spectrum for interaction of two delayed pulses of the form

31 $$E(t)\; = \;\left\{ {\begin{array}{*{20}l} {E_{0} \;sin[\omega t - \varphi _{1} ]\;sin^{2} [\frac{{\pi {\mkern 1mu} t}}{{T_{1} }}]\;} \hfill & {,t \in [0,\;T_{1} ]} \hfill \\ {0\;} \hfill & {,t \in [T_{1} ,T_{1} + T_{D} ]} \hfill \\ {E_{0} \;sin[\omega (t - T_{1} - T_{D} - \varphi _{2} )]\;sin^{2} [\frac{{\pi {\mkern 1mu} (t - T_{1} - T_{D} )}}{{T_{2} }}]\;} \hfill & {,t \in [T_{1} + T_{D} ,\;T_{2} ]} \hfill \\ \end{array} } \right.$$

is investigated. Here $T_D$ describes the time delay, $T_1\!\! =\!\! N_1 \frac{2\pi }{\omega }$, $T_2 \!\!=\!\! N_2 \frac{2\pi }{\omega }$, $\varphi _1 \!\!= \!\! \pi (N_1-\frac{1}{2})$, $\varphi _2 \!\!= \!\! \pi (N_2-\frac{1}{2}))$ and $N_1, N_2$ characterize the number of cycles for pulse 1 and 2. The individual pulse durations are sufficiently short, so that electrons are emitted in a wider energy range. Assuming that the electrons are released independently in each pulse - implying that the first wave packet is not affected by the second pulse- and that they overlap in energy, the interference pattern can be understood by modelling the electron wave packets by plane waves. During the first pulse, a wave packet with energy E is created at time $t'$ which develops to time *t* according to $a_1(E) e^{-iE(t-t')}$ with amplitude $a_1$. At creation time $t'$ the system - initially in its ground state with energy $E_i$ - contributes a phase factor $e^{-iE_i t'}$ to the wave packet. Adding this phase results in:

32a $$\begin{aligned} A_1 \; = \; a_1(E) \; e^{-iE(t-t') -iE_it'} \end{aligned}$$

The time delay is sufficiently short, so that the phase accumulation due to the Coulomb force in $[T_1, \! T_1+T_D]$ can be disregarded. In the second pulse, the electron is released at $t_2 = T_1 +T_D +t'$, and the phase of the ground state develops according $e^{-iE_i(T_1+T_D +t')}$ , giving :

32b $$\begin{aligned} A_2 \; = \; a_2(E) \; e^{-iE(t -T_1 -T_D-t') -iE_i(T_1+T_D+t')} \end{aligned}$$

The two wave packets overlap in energy. Superposing $A_1$ and $A_2$ and evaluating the squared modulus gives for the ionization spectrum:

33 $$\begin{aligned} \frac{dP}{dE} \; \sim \; \left[ A_1 +A_2 \right] \left[ A_1 +A_2 \right] ^* = | a_1|^2 + |a_2|^2 + 2 |a_1 a_2| \; \, cos \left[ (E-E_i)(T_1+T_D) \right] \end{aligned}$$

The maxima and minima are determined through the argument of the cosine function

34 $$\begin{aligned} E_{min} = E_i + \frac{\pi }{T_1+T_D}\,(2n+1),\quad E_{max} = E_i + \frac{\pi }{T_1+T_D}\,(2n), \quad n=0,1,2 \,\dots \end{aligned}$$

and are independent of the electric field strength. Figure 7 shows the ionization spectrum after interaction with two delayed attosecond pulses for two different field amplitudes. The electric field has the form of (31) with $\omega =2 \text { and } E_0=1$ (black curve) and $E_0=2$ (red curve). Each pulse consists of two cycles and the time delay is a whole oscillation period, $T_D = \pi = \frac{2\pi }{\omega }$. By way of illustration, pursuant to (34), the maxima are incorporated and represented by dashed vertical lines. The gap is given by $2\pi /(T_1+T_D)$ and provides an excellent correspondence of the modulation pattern with the predictions from equation (34). When $E_0=2$, the ionization probability is greatly enhanced. For both field strengths, maxima and minima are shown at the same position and fit surprisingly well with (34). The agreement of the interference structure with this plane wave approximation suggests the follwoing picture - at least in the strong field limit:

- The Coulomb interaction plays only a minor role; the interference structure can be described by plane waves.
- The wave packet produced by the first pulse is hardly affected by the second pulse (single photon regime). The oscillations in the energy spectrum can be seen as amplitudes and phase dependent overlays of the individual wave packets.
- Vice versa, the time delay of the pulses can be reconstructed from the interference patterns, while the height of the peaks scale with the field strength of the pulse.

## Conclusion

The aim of this article was to realize a TDSE solution with asymptotic boundary conditions based on $L^2$ functions which minimize reflections. These boundary conditions were formulated as outgoing waves based on analytical functions of the J-Matrix scattering theory. The method presented herein is competitive with other techniques like B-Spline or finite difference procedures and gives consistent results.

- The $L^2$ approach makes explicit use of the known analytic asymptotic solutions, which are fitted to the coupled differential equation system of the TDSE. The numerical implementation is significantly more efficient: at every time step the Hamilton matrix is only sparsely occupied. The affected matrix elements can be calculated a priori and standard procedures can be implemented for the time propagation.
- The determination of an optimal value for minimizing reflections can be realized adaptively in every time step, but does require a higher effort to calculate the Pollaczek functions.
- Physically relevant observables can be extracted by projecting the time dependent wave function onto analytically known Coulomb functions of the J-Matrix scattering theory.

A priori, it is to be expected that, with an expansion according to $L^2$ functions, this basis will describe the underlying phenomena well such as multiphoton ionization or above-treshold ionization in the weak field area which is also accessable via perturbation theory. A further aim of this article is to explicate the quality of $L^2$ expansion in areas in which a perturbation theoretical treatment no longer applies, i.e. in areas, where the strength of external fields become similar to or stronger than the inner atomic fields. The results can be summed up as follows:

- The method presented in this article allows one to describe ionization phenomena which are no longer accessible according to perturbation theory. The findings compare well with other independent methods.
- For short times, there is an excellent congruence with other independent approaches such as the B-spline and short time Magnus approximation.
- Interference structures in the ionization spectrum, based on delayed attosecond pulses, are successfully reproduced.

Only a small proportion of processes in attosecond fields were examined in this article. The method discussed here is well suited to describing other interesting phenomena:

- Description of the energy spectra, when phase effects of the external few cycle field are considered (Carrier Envelope Phase).
- Description of atomic systems within the single active electron approach. There the action with the core is incorporated through an effective potential, which in many cases contains a screened Coulomb potential and sums of exponentials combined with powers in the radial coordinate. Such potentials can be represented by Sturmian functions and can be implemented in the method presented herein.
- Description of recombination and generation of higher harmonics (HHG). This can be achieved through Fourier transformation of the time-dependent wave function.
- The modelling of the ionization dynamics when bound resonant intermediate states are involved.
- The method presented here can principially used for more exotic tailored external fields, e.g. vortex laser fields.
- The simulation of scattering effects in a diatomic molecule ion in the Born Oppenheimer approximation. This can be considered by making allowance for a further potential term in the TDSE.

## Data availability

The numerical datasets generated during the current study are available from the corresponding author upon reasonable request.
